# Structural characterization and comparative analysis of polymorphic forms of psilocin (4-hy­droxy-*N*,*N*-di­methyl­tryptamine)

**DOI:** 10.1107/S2056989024004201

**Published:** 2024-05-14

**Authors:** Matthias Zeller, Stephan Parent, Nate Schultheiss

**Affiliations:** a Purdue University, Department of Chemistry, 560 Oval Drive, West Lafayette, IN 47907-2084, USA; b Solsta Consulting LLC, 1200 Fawn Ridge Drive, West Lafayette, IN 47906, USA; c Canna-Chemistries LLC, 400 Main Street, Vincennes IN 47906, USA; University of Aberdeen, United Kingdom

**Keywords:** psilocin, psychedelic compounds, polymorphism, variable-temperature diffraction, crystal structure

## Abstract

The title compound, C_12_H_16_N_2_O, is a hy­droxy-substituted mono­amine alkaloid, and the primary metabolite of the naturally occurring psychedelic compound psilocybin. Crystalline forms of psilocin are known, but their characterization by single-crystal structure analysis is limited. Herein, two anhydrous polymorphic forms (I and II) of psilocin are described. The crystal structure of polymorphic Form I, in space group *P*2_1_/*c*, was first reported in 1974. Along with the redeterm­ination to modern standards and unambiguous location of the acidic H atom and variable-temperature single-crystal unit-cell determinations for Form I, the Form II polymorph of the title compound, which crystallizes in the monoclinic space group *P*2_1_/*n*, is described for the first time. The psilocin mol­ecules are present in both forms in their phenol–amine tautomeric forms (not resolved in the 1974 report). The mol­ecules in Forms I and II, however, feature different conformations of their *N*,*N*-dimethyl ethyl­ene substituent, with the N—C—C—C link in Form I being *trans* and in Form II being *gauche*, allowing the latter to bend back to the hydroxyl group of the same mol­ecule, leading to the formation of a strong intra­molecular O—H⋯N hydrogen bond between the hydroxyl moiety and ethyl­amino-nitro­gen group. In the extended structure of Form II, the mol­ecules form one-dimensional strands through N—H⋯O hydrogen bonds from the indole group to the oxygen atom of the hydroxyl moiety of an adjacent mol­ecule. Form II exhibits whole-mol­ecule disorder due to a pseudo-mirror operation, with an occupancy ratio of 0.689 (5):0.311 (5) for the two components. In contrast, Form I does not feature intra­molecular hydrogen bonds but forms a layered structure through inter­molecular N—H⋯O and O—H⋯N hydrogen bonds.

## Abstract 2

This study presents two anhydrous polymorphic forms of psilocin, detailing their crystal structures and hydrogen-bonding differences, with Form II introducing a novel conformation and whole-mol­ecule disorder.

## 1. Chemical context

Psychedelic-based therapies have garnered significant inter­est due to their potential for treating addiction, anxiety, depression, and post-traumatic stress disorder (PTSD) (Nichols, 2016 ▸). Currently, psilocybin {3-[2-(di­methyl­amino)­eth­yl]-1*H*-indol-4-yl di­hydrogen phosphate, C_12_H_17_N_2_O_4_P} is the most widely studied psychedelic compound for mental health conditions (Mitchell *et al.*, 2024 ▸). Upon entering the body, the prodrug psilocybin is de­phospho­rylated by the enzyme alkaline phosphatase, resulting in the active metabolite psilocin (4-hy­droxy-*N*,*N*-di­methyl­tryptamine, C_12_H_16_N_2_O). Psilocin is classified as a high-affinity agonist at serotonin 5-HT_2A_ receptors, which are responsible for producing the psychoactive effects within the body.

Crystalline materials derive their fundamental properties from the arrangement of individual mol­ecules within the solid. Consequently, polymorphic compounds often exhibit distinct physicochemical properties such as solubility, dissolution, stability, and melting point (Bernstein, 2002 ▸). Single-crystal structure analysis facilitates inspection of the mol­ecular connectivity and packing, which can lead to a more precise understanding of their structure–property relationships.

Psilocin was first chemically characterized by Albert Hofmann (Hofmann *et al.*, 1958 ▸), while a single-crystal structure of the title compound was later solved by Petcher *et al.* (1974 ▸) at room temperature in the space group *P*2_1_/*c*. The quality and resolution of the 1974 structure are, by modern standards, relatively poor. For example, the position of the acidic hydrogen atom was not resolved, leading to a questionable inter­pretation of the data, such as the possibility that the compound exists as a mixture of neutral mol­ecules and zwitterions. To get a better understanding of the structure of Form I, data were recollected at both room temperature and 150 K. Variable-temperature unit-cell determinations were also conducted, repeated every 20 K, starting from the complete dataset acquired at 150 K up to room temperature. This paper also describes for the first time the details of the crystal structure of a second polymorph (Form II) of psilocin, collected at room temperature in the space group *P*2_1_/*n* (preliminary data reported in a patent; Schultheiss *et al.*, 2022 ▸). 
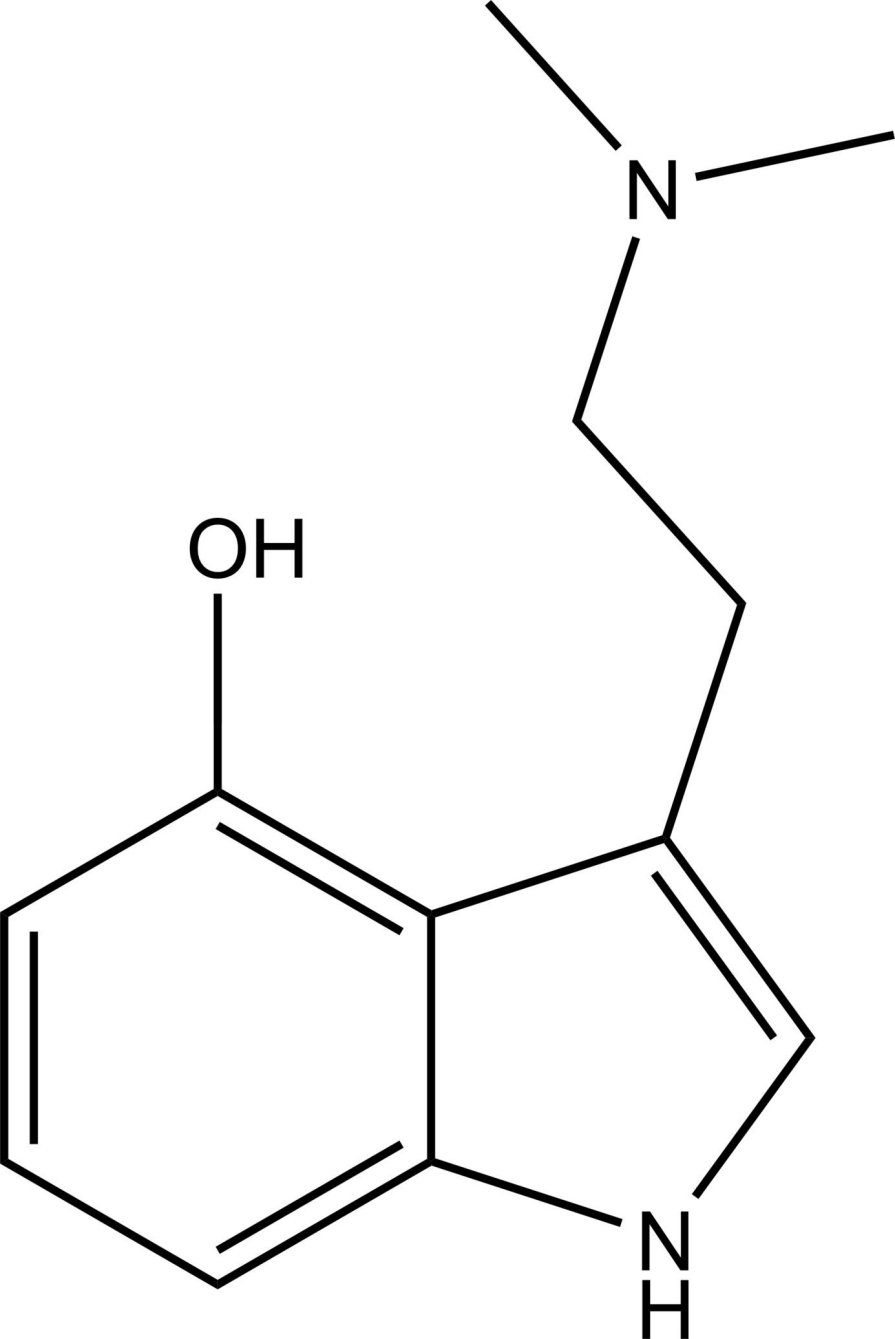


## 2. Structural commentary

Structural data for two polymorphic forms of psilocin, Forms I and II, were collected and analyzed. The two forms were obtained from different solvent mixtures: chloro­form/heptane for Form I and chloro­form for Form II. For Form II, the solution was acidified using HCl in attempts to form the psilocin hydro­chloride salt. A single crystal from the sample was identified and isolated; however, the bulk material was not suitable for additional analytical characterization. The exact mechanism that induces the formation of the different polymorphs remains unclear and requires additional exploration.

The two forms crystallize in different settings of the same centrosymmetric monoclinic space group, *P*2_1_/*c* and *P*2_1_/*n* for Forms I and II, respectively. Each form has one crystallographically independent mol­ecule (Z* = 1, *Z* = 4) and similar mol­ecular volume and density. Form I is more densely packed, with ρ = 1.202 rather than 1.190 g cm^−3^ for Form II (at room temperature). Although this difference could be attributed to the distinct mol­ecular conformations and packing between the forms (see below), we do not consider this difference to be significant.

**Figure 1:**
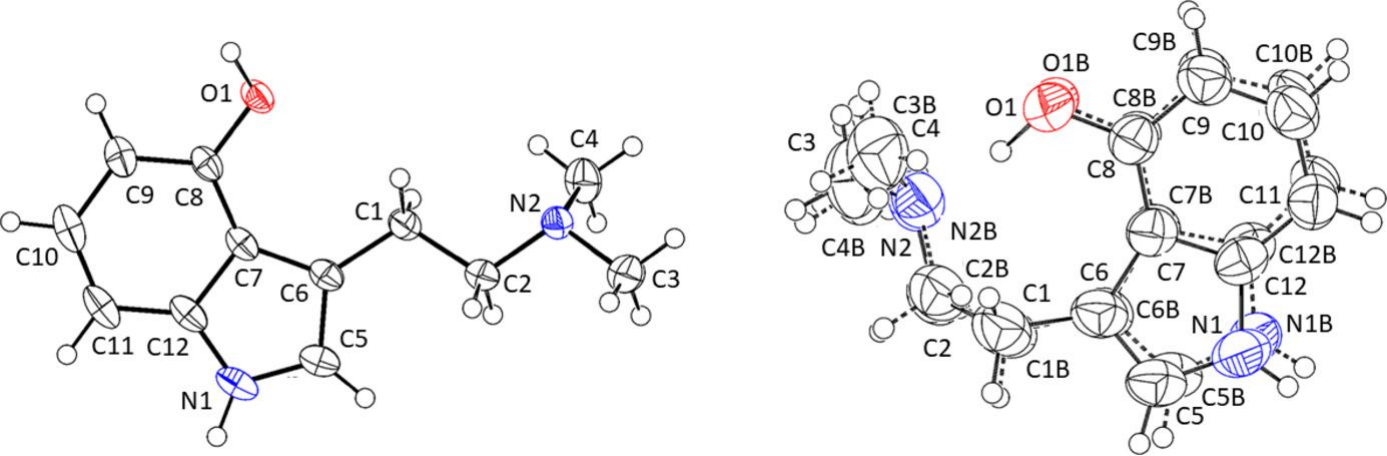
The mol­ecular structures of psilocin (Form I left, Form II right) drawn at the 50% probability level. The major components of disorder are shown.

Displacement ellipsoid plots of the mol­ecules for Form I and II are shown in Fig. 1 ▸. The mol­ecules in both forms share the almost planar 4-hy­droxy indole moiety. Bond lengths and angles in these moieties are unexceptional and as expected. The primary differences between the two forms lie in the mol­ecular conformations of the psilocin mol­ecules and the presence of disorder for Form II. Form I, previously described by Petcher and coworkers in 1974, exists in the crystal in its phenol–amine form, with the di­methyl­amine ethyl­ene pendant grouping rotated away from the phenol oxygen atom and hydrogen-bonded to the phenol hydroxyl group of a neighboring mol­ecule, forming centrosymmetric dimers (see *Supra­molecular features* section). In contrast, Form II establishes an intra­molecular O—H⋯N hydrogen bond, with the phenol O—H group bonded to the dimethyl amine of the same mol­ecule. Thus, the mol­ecules in the two forms differ by the torsion angles of the di­methyl­amine chain. For Form I, the C6—C1—C2—N2 unit is *trans* with a torsion angle of −172.27 (11)° [–170.10 (8)° at 150 K]. The same unit in Form II adopts a *gauche* conformation, with a torsion angle of −84.6 (6)° [83.2 (13)° for the minor disorder component] for the equivalent atoms.

Another aspect that differentiates the two polymorphs is the presence of whole-mol­ecule disorder in Form II. The entire mol­ecule is disordered across its average plane, induced by an inversion at the two ethyl­ene carbon atoms (C5 and C6). This is reflected in the already mentioned C6—C1—C2—N2 torsion angles, which are opposite in sign. The occupancy ratio refined to 0.689 (5):0.311 (5) (see the *Refinement* section for details). Consequently, the largest deviation between atom positions occurs at these two atoms (C1 and C2), but significant deviations are also observed for the disordered 4-hy­droxy indole and di­methyl­amine fragments [0.804 (8) Å for the O1⋯O1*B* distance, for example].

The intra­molecular O—H⋯N hydrogen bond is unaffected by the disorder; the acidic phenol proton is situated on the pseudo-mirror plane between the two moieties, maintaining the same position for both the major and minor moieties. As is typical for a strong hydrogen bond such as phenol-to-amine, the O—H distance is elongated beyond the standard hydroxyl O—H distance (0.82–0.84 Å for X-ray based data): see Tables 1 ▸–3 ▸ ▸ for all metric parameters. The refined O—H distances are 1.03 (2) Å for Form I [1.038 (19) Å at 150 K], and 1.08 (5) and 1.12 (5) Å for the major and minor moieties of Form II, respectively. These O—H bond lengths are slightly longer than those reported for similar compounds in the Cambridge Structural Database (CSD, 2023 version 5.45, including November 2023 update; Groom *et al.*, 2016 ▸): for 39 entries from 28 compounds featuring a phenol-to-di­methyl­amine hydrogen bond (excluding compounds where the O—H distance was constrained to a default value such as 0.82, 0.84 or 0.85 Å), an average O—H distance of 0.934 Å was reported. However, six of the 39 entries also feature O—H bond lengths greater than 1 Å, thus placing psilocin at the upper end of the observed range, although these values are not unprecedented. The H⋯N and O⋯N distances for Form I are 1.61 (2) and 2.6242 (14) Å [1.590 (19) and 2.6175 (10) Å at 150 K], and for Form II, they are 1.54 (5) and 2.584 (8) Å [1.43 (5) and 2.52 (2) Å for the minor moiety]. The average values from the CSD (for the same selection as for O—H distances) are 1.796 Å for H⋯N and 2.665 Å for the donor–acceptor distance, both significantly longer than the values found here, indicating that the elongated O—H distances in psilocin result from a very strong O—H⋯N hydrogen bond compared to other compounds with phenol to di­methyl­amine hydrogen bonds. This aligns with 4-hy­droxy indole being more acidic than phenol itself or alkyl-substituted phenols, which constitute the majority of the 28 compounds from the CSD. However, the potential inaccuracy of refined H atom positions from X-ray data, especially for older or lower quality/resolution data, should be considered when comparing the data observed here with those from the CSD.

In their previous description of the structure of Form I, Petcher *et al.* (1974 ▸) were unable to locate the acidic hydrogen atom and speculated that the compound likely exists as a mixture of neutral mol­ecules and zwitterions; that is, the phenol H atom might occasionally be transferred to the amine, forming a phenolate–ammonium proton-transfer complex. Proton transfer from a (relatively) strongly acidic phenol to an amine would not be unprecedented. However, observations of this in the solid state are, with one exception, limited to very acidic di- and tri­nitro-substituted phenolates (25 entries in the CSD, excluding metal-coordinated phenolates). The one exception is a 4-bromo substituted phenolate (Ghosh *et al.*, 2022 ▸). In this case, the proton transfer to the dimethyl amine is facilitated by a second N—H group (from a hydrazinyl fragment) also hydrogen-bonded to the phenolate, thus stabilizing the anionic charge. No cases of a partial proton transfer, with an equilibrium between O—H⋯N and N—H^+^⋯O^−^ states, have been reported for the solid state. With the new data now on hand, this can unequivocally be excluded as an option for psilocin. The electron densities of the acidic protons are well resolved for both polymorphic forms, and they are clearly associated with the phenol oxygen atom. The O—H bond lengths are elongated as expected for a strong hydrogen bond (as described above), but no split electron density or a full transfer of the proton is observed in either Form I (RT and 150 K) or in Form II.

**Figure 2:**
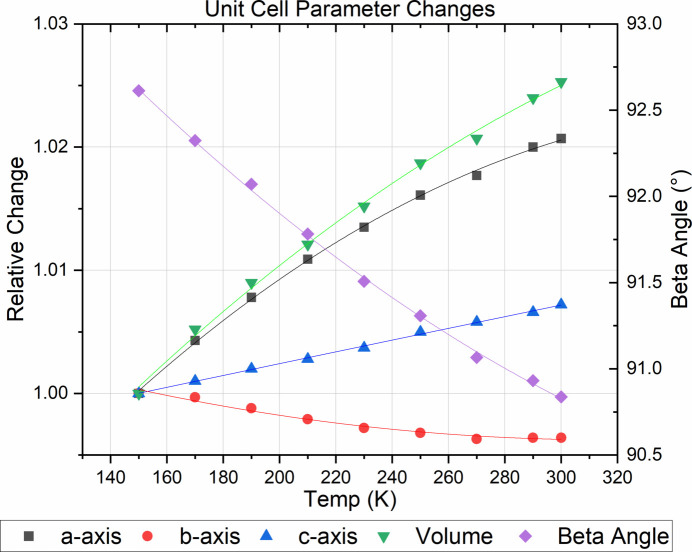
Variable-temperature unit-cell data of Form I from 150–300 K expressed as relative change compared to the 150 K values.

To unequivocally exclude the possibility of partial proton transfer for psilocin in the solid state, low-temperature data were also collected for the better-defined, non-disordered polymorph, Form I. The refinement against the data collected at 150 K showed no indication of partial or full proton transfer, with well-defined and localized electron densities for the acidic hydrogen atoms. Variable-temperature unit-cell data were collected as well, showing a steady change in all unit-cell parameters between 300 and 150 K, with no indications of the presence of a discontinuity or phase change, see Fig. 2 ▸ and supporting information. The *a*- and *c*-axes exhibit a steady decline on cooling, while the *b*-axis marginally increases. The largest relative changes are observed for the *a*-axis and the unit-cell volume, which shrink by ∼2 and 2.5%, respectively, upon cooling. The β angle increases from 90.8366 (24)° at 300 K to 92.6122 (19)° at 150 K (see supporting information). Overall, no unusual behavior is observed in the studied temperature range.

The closeness of the β angle to 90° allows for twinning by pseudo-merohedry. During the screening of crystals for data collection, twinning was indeed observed for some crystals (TWIN transformation matrix −1 0 0 / 0 −1 0 / 0 0 1, corresponding to twofold rotation around the *c*-axis). Pseudo-merohedry led to twinning by non-merohedry upon cooling. All data reported here were collected from a crystal that was not twinned.

## 3. Supra­molecular features

**Figure 3:**
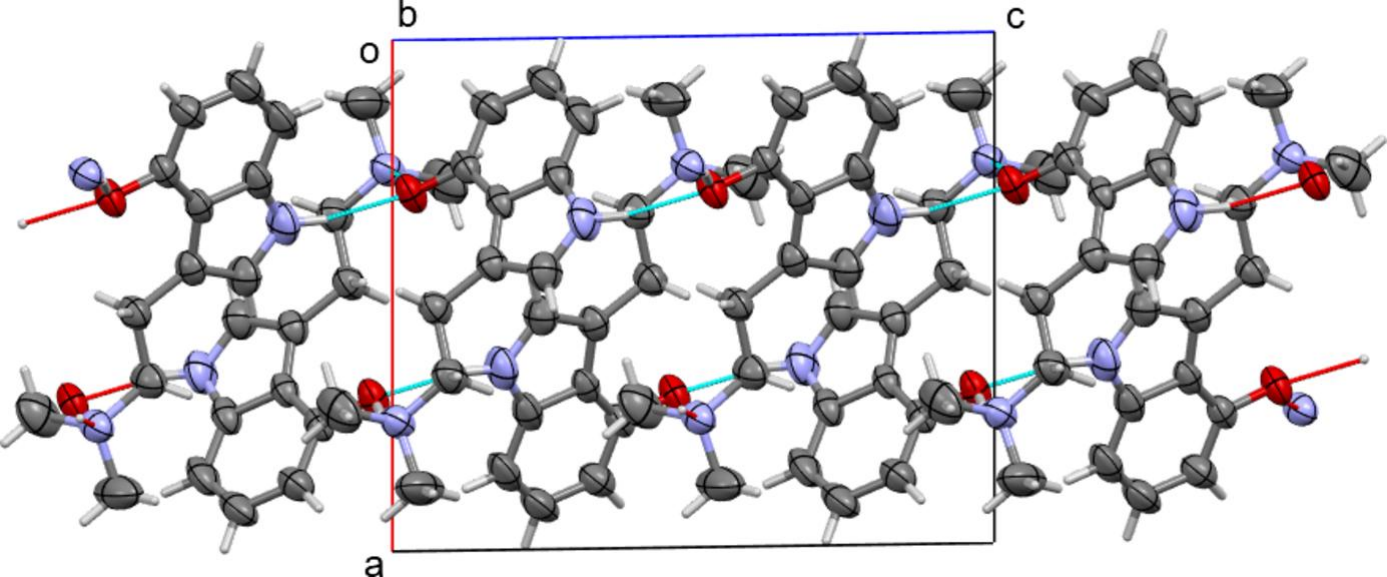
Inter­molecular N—H⋯O hydrogen bonding between mol­ecules of Form I.

**Figure 4:**
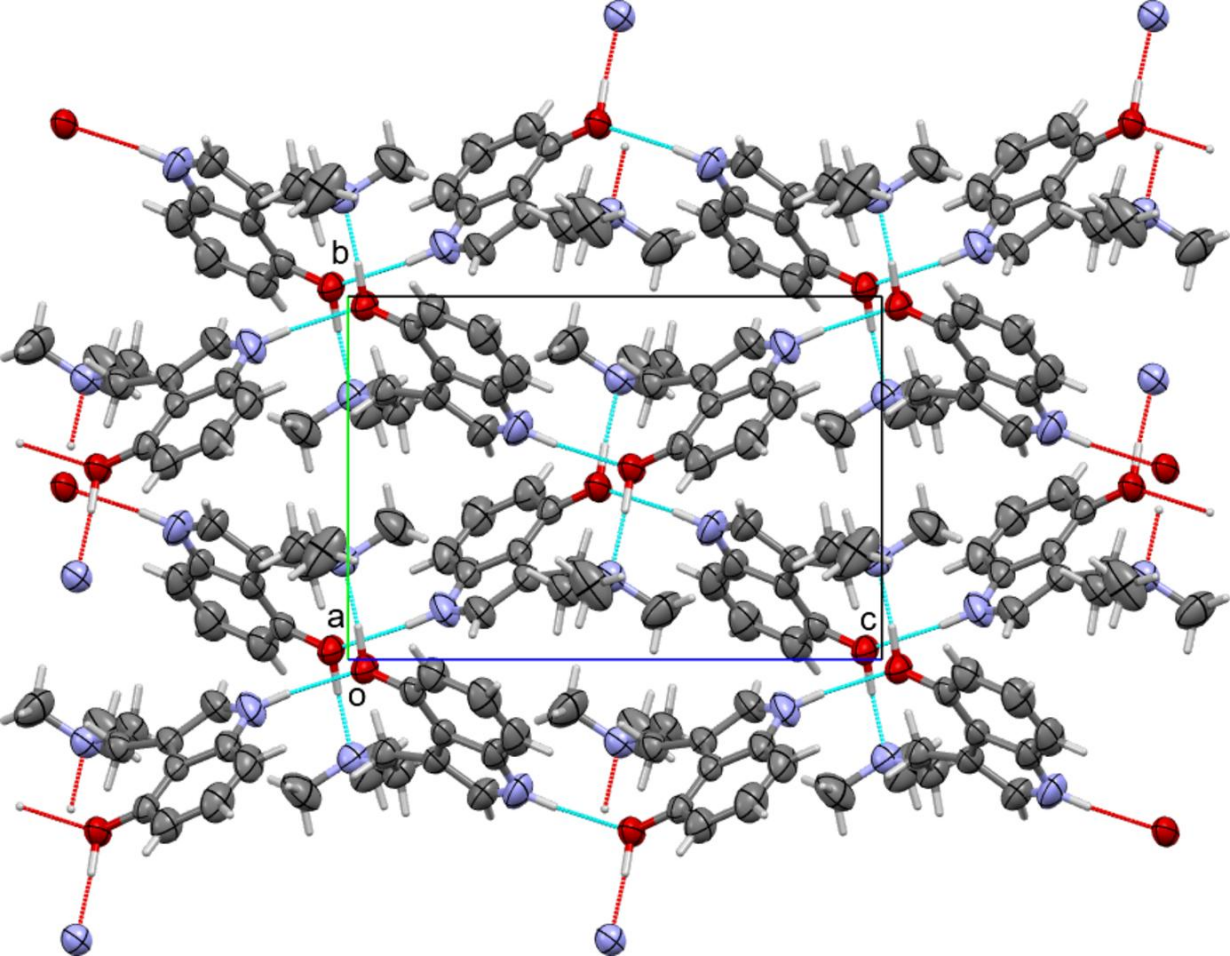
Inter­molecular N—H⋯O and O—H⋯N hydrogen bonding between mol­ecules of Form I resulting in two-dimensional layers.

The difference between Forms I and II of psilocin are based on mol­ecular conformations that lead to either intra- or inter­molecular O—H⋯N bonds. Consequently, inter­actions between neighboring mol­ecules also differ significantly between the two polymorphs. In Form I, all hydrogen-bonding inter­actions are inter­molecular. Two types of classical hydrogen bonds involving acidic protons are observed: phenol OH to amine, and indole N—H to phenol. The O—H⋯N hydrogen bonds connect pairs of mol­ecules into hydrogen-bonded dimers, see Fig. 3 ▸: mol­ecules are inversion-related [symmetry operator: (i) 1 − *x*, 1 − *y*, 1 − *z*]. The N—H⋯O hydrogen bonds operate perpendicular to the plane of the inversion dimers [symmetry operator: (ii) *x*, 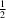 − *y*, 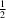 + *z*], thus connecting the mol­ecules to other dimers and creating infinite layers that extend perpendicular to the *a*-axis direction. Additional weak inter­actions enhance adhesion within the layers, such as a weak C—H⋯O inter­action originating from methyl­ene C atom C1, supporting the inversion dimer, and a C—H⋯π inter­action of a pyrrole H atom to a neighboring phenyl ring [C5—H5 to C7^iv^, symmetry operator: (iv) 1 − *x*, − 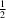 + *y*, 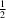 − *z*]. Perpendicular to the layers, along [100], no hydrogen bonds are realized, and no other directional inter­actions such as C—H⋯O, C—H⋯N, C—H⋯π, or π-stacking inter­actions are present, thus creating a distinctly two-dimensional layered packing arrangement, see Fig. 4 ▸.

**Figure 5:**
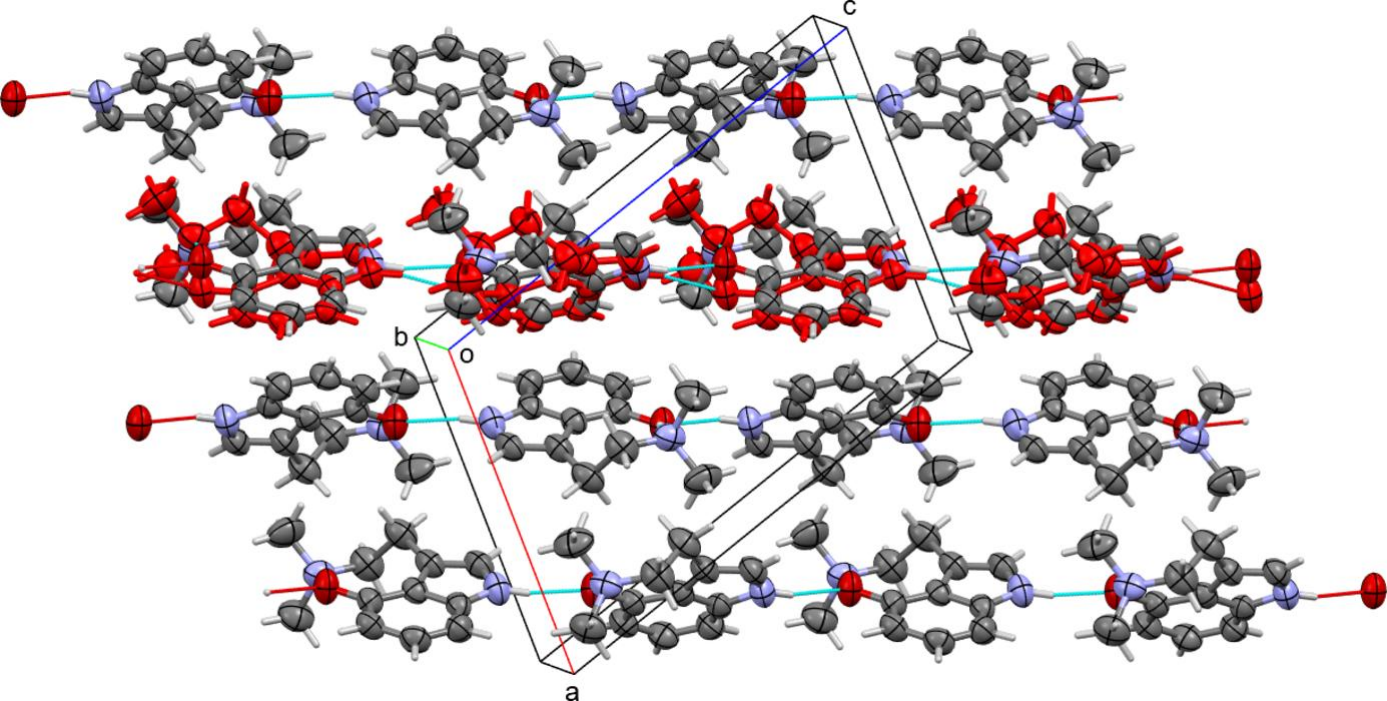
Hydrogen bonding in Form II with infinite chains along [101]. Minor moieties of the whole-mol­ecule disorder are shown for one strand in red. Inter­molecular N—H⋯O hydrogen bonds displayed in turquoise.

In Form II, the O—H⋯N hydrogen bond is intra­molecular, as described above. An inter­molecular indole N—H to phenol O bond is also present, as in Form I. This inter­action connects mol­ecules along the *ac*-diagonal (the [101] direction) into infinite chains. Parallel chains with opposite direction (inversion-related to the original chain) extend in parallel, thus creating an infinite array of N—H⋯O connected chains, see Fig. 5 ▸. Minor weak inter­actions connect neighboring chains. For the major disordered moiety, the most prominent inter­action is a C—H⋯π inter­action of a methyl group to C6 of a neighboring pyrrole ring (at 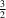 − *x*, − 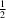 + *y*, 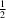 − *z*). For the minor moiety, a number of shorter-than-usual contacts involve close contacts between C atoms that are unlikely to be attractive inter­actions, like a 3.259 Å contact between methyl C atoms C3*B* and C3*B* ^v^ [symmetry operator: (v) 2 − *x*, 1 − *y*, 1 − *z*]. The most unfavorable inter­actions are present only between neighboring mol­ecules of minor moieties, providing an explanation for why less than 50% of mol­ecules are in the minor moiety orientation, as more than 50% occupancy would invariably lead to close and unfavorable contacts. The actual approximate 2:1 occupancy ratio [refined: 0.689 (5) to 0.311 (5)] avoids those close contacts.

## 4. Database survey

A search of the CSD revealed one entry of 4-hy­droxy-*N*,*N*-di­methyl­tryptamine (psilocin) and one entry of a structurally very similar mol­ecule, 5-hy­droxy-*N*,*N*-di­methyl­tryptamine (bufotenine). The Form I (*P*2_1_/*c*) polymorph of 4-hy­droxy-*N*,*N*-di­methyl­tryptamine, with data collected at room temperature, was reported in 1974 (CSD refcode PSILIN; Petcher *et al.*, 1974 ▸). The crystal structure of 5-hy­droxy-*N*,*N*-di­methyl­tryptamine, also collected at room temperature, was reported in 1972 (CSD refcode BUFTEN; Falkenberg 1972 ▸). In this case, the mol­ecule possesses two hydrogen-bond donors/acceptors; however, moving the hydroxyl group from the 4- to the 5-position results in one-dimensional strands formed through a series of O—H⋯N hydrogen bonds (O⋯N = 2.719 Å) from the hydroxyl group to the ethyl­amino nitro­gen atom. The indole hydrogen-bond donor and hydroxyl oxygen hydrogen-bond acceptor do not participate significantly in the overall structure. A series of results featuring the 4-hy­droxy-*N*,*N*-di­alkyl­tryptamine structural backbone were identified. In each case, when the tryptamine mol­ecule reacts with iodo­methane (MeI), the ethyl­amino group is alkyl­ated, resulting in the formation of iodide salts (CSD refcodes EDOYIJ, EDOYUV, EDOZIK, XUXFAA; Glatfelter *et al.*, 2022 ▸; Chadeayne *et al.*, 2020*b* ▸). Similarly, when 4-hy­droxy-*N*,*N*-di­alkyl­tryptamine reacts with fumaric acid, the ethyl­amino group protonates, resulting in fumarate salts (CSD refcodes RONSUL, TUFQAP, WUCGAF; Chadeayne *et al.*, 2019*a* ▸,*b* ▸, 2020*a* ▸).

## 5. Synthesis and crystallization

Crystallization of Form I: 44.6 mg of psilocin (Cayman Chemical) was dissolved in a 20-ml solvent mixture of chloro­form (Supelco) and heptane (Sigma-Aldrich) in a 1:4 volume-to-volume ratio. The clear solution obtained was left to evaporate at ambient conditions until dry, resulting in the formation of rosettes of crystalline plates.

Crystallization of Form II: a suspension of 129.4 mg of psilocin (Cayman Chemical) in 8 ml of chloro­form (Macron Chemicals) was briefly sonicated in the presence of activated charcoal. After sonication, the mixture was filtered through a 0.2 µm nylon filter to remove particulates. The resulting clear solution was then acidified by adding 52 ml of 37% HCl (Sigma-Aldrich) over dry ice. The mixture was left to stand in the freezer for one day to facilitate phase separation. Subsequently, the upper layer was deca­nted from the biphasic mixture. The supernatant was partially evaporated under a stream of dry nitro­gen at room temperature until lamellar plates began to form.

## 6. Refinement

Crystal data, data collection and structure refinement details are summarized in Table 4 ▸. For both Forms I and II, the carbon-bound hydrogen atoms were refined isotropically at calculated positions using a riding model. The methyl H atoms were allowed to rotate but not to tip to best fit the experimental electron density. The positions and displacement parameters of acidic H atoms (O—H and N—H) were freely refined. The displacement parameter of the acidic phenol H atom of Form II was freely refined. *U*_iso_ values were constrained to 1.5 times the *U*_eq_ of their pivot atoms for the other acidic H atoms and methyl groups and 1.2 times for all other hydrogen atoms.

For Form II, disorder was observed and refined. A rotation of the ethyl­ene bridge connecting the dimethyl amino group to the indole ring system induces whole-mol­ecule disorder. The two disordered moieties were restrained to have similar geometries [SAME command in *SHELXL* (Sheldrick, 2015*b* ▸)]. The indene fragment of the minor moiety, including the directly adjacent C and O atoms, was restrained to be close to planar (FLAT command). The acidic phenol H atom was excluded from the disorder, and its position and displacement parameter were freely refined. The *U*_ij_ components of ADPs for disordered atoms closer to each other than 2.0 Å were restrained to be similar. Subject to these conditions, the occupancy ratio refined to 0.689 (5) to 0.311 (5).

For the variable temperature unit cell measurements, a colorless, plate-shaped crystal of Form I (the same as used for the full data collections) was mounted on a Mitegen micromesh mount in a random orientation. Data were collected on a Bruker AXS D8 Quest three-circle diffractometer with a fine-focus sealed tube X-ray source, using a Triumph curved graphite crystal as monochromator and a PhotonII charge-integrating pixel array (CPAD) detector. The diffractometer used Mo *K_α_* radiation (λ = 0.71073Å). The crystal was initially shock-cooled to 150 K, at which temperature a full dataset was collected. The temperature was then increased at a rate of 6° per minute to the next target temperature. After a waiting period of 15 minutes for temperature equilibration, a 180° φ scan (‘Fast Scan’) was collected (5 cm detector-to-crystal distance, 2θ = 0°, shutterless continuous mode, 5 seconds exposure time per read out every 1°). The last 120 of the 180 frames of each run were integrated using *SAINT V8.40B* (Bruker, 2020 ▸). The results (unit-cell parameters, orientation matrices, and correction parameters) were reimported, and the integration was repeated once. The procedure was repeated every 20 K, and the unit-cell data were obtained from the integration files (p4p files).

## Supplementary Material

Crystal structure: contains datablock(s) Form_II_293K, Form_I_150K, Form_I_300K, global. DOI: 10.1107/S2056989024004201/hb8096sup1.cif

Structure factors: contains datablock(s) Form_II_293K. DOI: 10.1107/S2056989024004201/hb8096Form_II_293Ksup2.hkl

Structure factors: contains datablock(s) Form_I_150K. DOI: 10.1107/S2056989024004201/hb8096Form_I_150Ksup3.hkl

Structure factors: contains datablock(s) Form_I_300K. DOI: 10.1107/S2056989024004201/hb8096Form_I_300Ksup4.hkl

Supporting information file. DOI: 10.1107/S2056989024004201/hb8096Form_II_293Ksup5.cml

CCDC references: 2353848, 2353847, 2353846

Additional supporting information:  crystallographic information; 3D view; checkCIF report

## Figures and Tables

**Table 1:** Hydrogen-bond geometry (Å, °) for Form I at 150 K[Chem scheme1]

*D*—H⋯*A*	*D*—H	H⋯*A*	*D*⋯*A*	*D*—H⋯*A*
O1—H1*O*⋯N2^i^	1.038 (19)	1.590 (19)	2.6175 (10)	169.5 (16)
N1—H1*N*⋯O1^ii^	0.913 (16)	1.995 (17)	2.8867 (11)	165.0 (15)

Symmetry codes: (i) 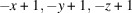 ; (ii) 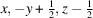 .

**Table 2:** Hydrogen-bond geometry (Å, °) for Form I at 300 K[Chem scheme1]

*D*—H⋯*A*	*D*—H	H⋯*A*	*D*⋯*A*	*D*—H⋯*A*
O1—H1*O*⋯N2^i^	1.03 (2)	1.61 (2)	2.6242 (14)	166.6 (18)
N1—H1*N*⋯O1^ii^	0.92 (2)	1.99 (2)	2.8944 (14)	167.9 (18)

Symmetry codes: (i) 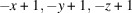 ; (ii) 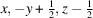 .

**Table 3:** Hydrogen-bond geometry (Å, °) for Form II at 293 K[Chem scheme1]

*D*—H⋯*A*	*D*—H	H⋯*A*	*D*⋯*A*	*D*—H⋯*A*
N1—H1*N*⋯O1^i^	0.86	1.99	2.823 (8)	164
N1*B*—H1*NB*⋯O1*B* ^i^	0.86	2.14	2.984 (18)	169
O1—H1⋯N2	1.08 (5)	1.54 (5)	2.584 (8)	161 (4)
O1*B*—H1⋯N2*B*	1.12 (5)	1.43 (5)	2.52 (2)	162 (4)

Symmetry code: (i) 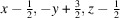 .

**Table 4:** Experimental details

	Form I at 150 K	Form I at 300 K	Form II at 293 K
Crystal data			
Chemical formula	C_12_H_16_N_2_O	C_12_H_16_N_2_O	C_12_H_16_N_2_O
*M*_r_	204.27	204.27	204.27
Crystal system, space group	Monoclinic, *P*2_1_/*c*	Monoclinic, *P*2_1_/*c*	Monoclinic, *P*2_1_/*n*
Temperature (K)	150	300	293
*a*, *b*, *c* (Å)	10.4041 (4), 8.5286 (3), 12.4087 (4)	10.6228 (5), 8.4984 (4), 12.5073 (5)	9.5331 (7), 8.9358 (3), 14.0279 (7)
β (°)	92.5663 (19)	90.807 (2)	107.490 (7)
*V* (Å^3^)	1099.95 (7)	1129.01 (9)	1139.73 (11)
*Z*	4	4	4
Radiation type	Mo *K*α	Mo *K*α	Cu *K*α
μ (mm^−1^)	0.08	0.08	0.61
Crystal size (mm)	0.43 × 0.29 × 0.06	0.43 × 0.29 × 0.06	0.37 × 0.16 × 0.04

Data collection			
Diffractometer	Bruker AXS D8 Quest	Bruker AXS D8 Quest	SuperNova, Single source at offset, Pilatus 200/300K
Absorption correction	Multi-scan (*SADABS* ; Krause *et al.*, 2015 ▸)	Multi-scan (*SADABS*; Krause *et al.*, 2015 ▸)	Multi-scan (*CrysAlis PRO*; Rigaku OD, 2015 ▸)
*T*_min_, *T*_max_	0.663, 0.747	0.663, 0.747	0.675, 1.000
No. of measured, independent and observed [*I* > 2σ(*I*)] reflections	33575, 4199, 3116	34680, 4323, 2421	4929, 2329, 1754
*R*_int_	0.051	0.066	0.019
(sin θ/λ)_max_ (Å^−1^)	0.770	0.771	0.634

Refinement			
*R*[*F* ^2^ > 2σ(*F* ^2^)], *wR*(*F* ^2^), *S*	0.046, 0.138, 1.05	0.052, 0.172, 1.03	0.065, 0.221, 1.13
No. of reflections	4199	4323	2329
No. of parameters	146	146	280
No. of restraints	0	0	532
H-atom treatment	H atoms treated by a mixture of independent and constrained refinement	H atoms treated by a mixture of independent and constrained refinement	H atoms treated by a mixture of independent and constrained refinement
Δρ_max_, Δρ_min_ (e Å^−3^)	0.35, −0.24	0.21, −0.19	0.18, −0.16

Computer programs: *CrysAlis PRO* (Rigaku OD, 2015 ▸), *APEX4* (Bruker, 2022 ▸), *SAINT* (Bruker, 2020 ▸), *SHELXT* (Sheldrick, 2015*a* ▸), *SHELXL2019/2* (Sheldrick, 2015*b* ▸), *ShelXle* (Hübschle *et al.*, 2011 ▸), *Mercury* (Macrae *et al.*, 2020 ▸) and *publCIF* (Westrip, 2013 ▸).

## supplementary crystallographic information

### 3-[2-(Dimethylamino)ethyl]-1H-indol-4-ol (Form_II_293K) Crystal data

**Table:**

C_12_H_16_N_2_O	*F*(000) = 440
*M**_r_* = 204.27	*D*_x_ = 1.190 Mg m^−^^3^
Monoclinic, *P*2_1_/*n*	Cu *K*α radiation, λ = 1.54184 Å
*a* = 9.5331 (7) Å	Cell parameters from 2141 reflections
*b* = 8.9358 (3) Å	θ = 4.9–77.1°
*c* = 14.0279 (7) Å	µ = 0.61 mm^−^^1^
β = 107.490 (7)°	*T* = 293 K
*V* = 1139.73 (11) Å^3^	Plate, colourless
*Z* = 4	0.37 × 0.16 × 0.04 mm

### 3-[2-(Dimethylamino)ethyl]-1H-indol-4-ol (Form_II_293K) Data collection

**Table:**

SuperNova, Single source at offset, Pilatus 200/300K diffractometer	1754 reflections with *I* > 2σ(*I*)
Radiation source: micro-focus sealed X-ray tube, SuperNova (Cu) X-ray Source	*R*_int_ = 0.019
ω scans	θ_max_ = 78.0°, θ_min_ = 5.0°
Absorption correction: multi-scan (CrysAlisPro; Rigaku OD, 2015)	*h* = −11→8
*T*_min_ = 0.675, *T*_max_ = 1.000	*k* = −11→11
4929 measured reflections	*l* = −17→17
2329 independent reflections	

### 3-[2-(Dimethylamino)ethyl]-1H-indol-4-ol (Form_II_293K) Refinement

**Table:**

Refinement on *F*^2^	Primary atom site location: dual
Least-squares matrix: full	Secondary atom site location: difference Fourier map
*R*[*F*^2^ > 2σ(*F*^2^)] = 0.065	Hydrogen site location: mixed
*wR*(*F*^2^) = 0.221	H atoms treated by a mixture of independent and constrained refinement
*S* = 1.13	*w* = 1/[σ^2^(*F*_o_^2^) + (0.1062*P*)^2^ + 0.3139*P*] where *P* = (*F*_o_^2^ + 2*F*_c_^2^)/3
2329 reflections	(Δ/σ)_max_ < 0.001
280 parameters	Δρ_max_ = 0.18 e Å^−^^3^
532 restraints	Δρ_min_ = −0.16 e Å^−^^3^

### 3-[2-(Dimethylamino)ethyl]-1H-indol-4-ol (Form_II_293K) Special details

**Table:**

Geometry. All esds (except the esd in the dihedral angle between two l.s. planes) are estimated using the full covariance matrix. The cell esds are taken into account individually in the estimation of esds in distances, angles and torsion angles; correlations between esds in cell parameters are only used when they are defined by crystal symmetry. An approximate (isotropic) treatment of cell esds is used for estimating esds involving l.s. planes.
Refinement. Rotation of the ethylene bridge connecting the dimethyl amino group induces whole molecule disorder. The two disordered moieties were restrained to have similar geometries. The indene fragment of the minor moiety including the directly adjacent C and O atoms was restrained to be close to planar. The acidic phenol H atom was excluded from the disorder and its position and thermal parameter were freely refined. Uij components of ADPs for disordered atoms closer to each other than 2.0 Angstrom were restrained to be similar. Subject to these conditions the occupancy ratio refined to 0.689 (5) to 0.311 (5).

### 3-[2-(Dimethylamino)ethyl]-1H-indol-4-ol (Form_II_293K) Fractional atomic coordinates and isotropic or equivalent isotropic displacement parameters (Å^2^)

**Table:**

	*x*	*y*	*z*	*U*_iso_*/*U*_eq_	Occ. (<1)
H1	0.629 (5)	0.548 (5)	0.379 (3)	0.120 (13)*	
C1	0.6049 (6)	0.4321 (5)	0.1961 (3)	0.0786 (12)	0.689 (5)
H1A	0.701941	0.477062	0.218344	0.094*	0.689 (5)
H1B	0.598839	0.374580	0.136326	0.094*	0.689 (5)
C2	0.5891 (7)	0.3248 (5)	0.2772 (3)	0.0793 (11)	0.689 (5)
H2A	0.485373	0.309139	0.268700	0.095*	0.689 (5)
H2B	0.631533	0.228959	0.268722	0.095*	0.689 (5)
N2	0.6602 (6)	0.3790 (7)	0.3790 (4)	0.0700 (13)	0.689 (5)
C3	0.8215 (7)	0.3657 (8)	0.4111 (6)	0.0991 (18)	0.689 (5)
H3A	0.848490	0.262228	0.410669	0.149*	0.689 (5)
H3B	0.860680	0.405160	0.477477	0.149*	0.689 (5)
H3C	0.860535	0.420937	0.366147	0.149*	0.689 (5)
C4	0.6000 (8)	0.3008 (7)	0.4497 (5)	0.0880 (16)	0.689 (5)
H4A	0.496721	0.321472	0.433727	0.132*	0.689 (5)
H4B	0.649220	0.334574	0.516344	0.132*	0.689 (5)
H4C	0.614668	0.195002	0.445389	0.132*	0.689 (5)
N1	0.3190 (10)	0.6949 (9)	0.0677 (6)	0.0731 (15)	0.689 (5)
H1N	0.253727	0.724472	0.014198	0.110*	0.689 (5)
C5	0.3974 (7)	0.5670 (8)	0.0772 (5)	0.0692 (15)	0.689 (5)
H5	0.387132	0.496668	0.026696	0.083*	0.689 (5)
C6	0.4918 (6)	0.5555 (6)	0.1692 (4)	0.0646 (11)	0.689 (5)
C7	0.4692 (9)	0.6852 (8)	0.2248 (5)	0.0557 (14)	0.689 (5)
C8	0.5353 (6)	0.7405 (7)	0.3216 (4)	0.0573 (12)	0.689 (5)
C9	0.4903 (8)	0.8781 (8)	0.3460 (6)	0.0651 (16)	0.689 (5)
H9	0.533483	0.918116	0.409263	0.078*	0.689 (5)
C10	0.3792 (13)	0.9580 (11)	0.2755 (6)	0.0687 (18)	0.689 (5)
H10	0.346823	1.047946	0.294841	0.082*	0.689 (5)
C11	0.3173 (13)	0.9095 (11)	0.1803 (6)	0.0683 (17)	0.689 (5)
H11	0.249568	0.967390	0.133219	0.082*	0.689 (5)
C12	0.3599 (13)	0.7700 (10)	0.1570 (6)	0.0570 (13)	0.689 (5)
O1	0.6453 (5)	0.6668 (4)	0.3906 (3)	0.0720 (9)	0.689 (5)
C1B	0.5012 (11)	0.3867 (10)	0.2090 (7)	0.078 (2)	0.311 (5)
H1C	0.478936	0.323039	0.150278	0.094*	0.311 (5)
H1D	0.446315	0.348423	0.251780	0.094*	0.311 (5)
C2B	0.6645 (12)	0.3715 (13)	0.2644 (7)	0.079 (2)	0.311 (5)
H2C	0.696312	0.270113	0.257242	0.095*	0.311 (5)
H2D	0.720114	0.439026	0.235409	0.095*	0.311 (5)
N2B	0.6939 (15)	0.4062 (19)	0.3706 (9)	0.073 (3)	0.311 (5)
C3B	0.8539 (14)	0.443 (2)	0.4141 (14)	0.105 (4)	0.311 (5)
H3D	0.911578	0.355453	0.411664	0.158*	0.311 (5)
H3E	0.872839	0.473878	0.482286	0.158*	0.311 (5)
H3F	0.879671	0.521794	0.376268	0.158*	0.311 (5)
C4B	0.6546 (16)	0.2889 (18)	0.4287 (12)	0.096 (3)	0.311 (5)
H4D	0.549796	0.276267	0.407503	0.143*	0.311 (5)
H4E	0.687337	0.315529	0.498172	0.143*	0.311 (5)
H4F	0.700690	0.197023	0.418951	0.143*	0.311 (5)
N1B	0.306 (2)	0.722 (2)	0.0774 (13)	0.068 (2)	0.311 (5)
H1NB	0.248617	0.762923	0.024437	0.102*	0.311 (5)
C5B	0.3611 (15)	0.5824 (19)	0.0815 (11)	0.065 (2)	0.311 (5)
H5B	0.343952	0.519523	0.026288	0.078*	0.311 (5)
C6B	0.4454 (12)	0.5433 (12)	0.1756 (10)	0.067 (2)	0.311 (5)
C7B	0.446 (2)	0.6822 (18)	0.2322 (11)	0.055 (2)	0.311 (5)
C8B	0.4966 (13)	0.7291 (15)	0.3325 (9)	0.056 (2)	0.311 (5)
C9B	0.4612 (18)	0.8680 (16)	0.3603 (12)	0.058 (2)	0.311 (5)
H9B	0.499146	0.895479	0.427087	0.070*	0.311 (5)
C10B	0.374 (3)	0.968 (2)	0.2955 (13)	0.059 (2)	0.311 (5)
H10B	0.357485	1.063956	0.316512	0.071*	0.311 (5)
C11B	0.312 (3)	0.924 (2)	0.2009 (13)	0.059 (2)	0.311 (5)
H11B	0.241889	0.984553	0.156808	0.071*	0.311 (5)
C12B	0.352 (3)	0.788 (2)	0.1687 (14)	0.058 (2)	0.311 (5)
O1B	0.5739 (11)	0.6420 (9)	0.4069 (5)	0.076 (2)	0.311 (5)

### 3-[2-(Dimethylamino)ethyl]-1H-indol-4-ol (Form_II_293K) Atomic displacement parameters (Å^2^)

**Table:**

	*U*^11^	*U*^22^	*U*^33^	*U*^12^	*U*^13^	*U*^23^
C1	0.106 (3)	0.067 (2)	0.072 (2)	0.004 (2)	0.041 (2)	−0.0041 (17)
C2	0.105 (3)	0.055 (2)	0.081 (2)	0.003 (2)	0.033 (2)	−0.0001 (17)
N2	0.078 (3)	0.059 (3)	0.080 (2)	0.0124 (19)	0.0338 (19)	0.0092 (18)
C3	0.072 (3)	0.097 (4)	0.134 (4)	0.026 (3)	0.039 (3)	0.019 (4)
C4	0.104 (4)	0.076 (3)	0.094 (3)	−0.002 (3)	0.045 (3)	0.015 (2)
N1	0.084 (3)	0.078 (4)	0.050 (2)	−0.007 (2)	0.0099 (18)	−0.004 (2)
C5	0.084 (3)	0.072 (3)	0.0559 (19)	−0.008 (2)	0.028 (2)	−0.0104 (18)
C6	0.082 (3)	0.0618 (19)	0.0557 (17)	−0.0055 (19)	0.028 (2)	−0.0054 (14)
C7	0.064 (3)	0.0537 (18)	0.0522 (19)	−0.0065 (17)	0.0216 (17)	0.0023 (15)
C8	0.064 (3)	0.0563 (19)	0.0525 (19)	−0.0007 (19)	0.0193 (18)	0.0034 (15)
C9	0.081 (3)	0.060 (2)	0.059 (3)	0.001 (2)	0.028 (2)	−0.0028 (18)
C10	0.078 (2)	0.063 (3)	0.072 (4)	0.009 (2)	0.032 (3)	−0.004 (3)
C11	0.069 (2)	0.070 (3)	0.066 (3)	0.011 (2)	0.020 (3)	0.005 (2)
C12	0.059 (2)	0.063 (3)	0.049 (2)	−0.009 (2)	0.0173 (19)	0.0030 (19)
O1	0.083 (2)	0.0611 (17)	0.0585 (17)	0.0045 (16)	0.0007 (15)	−0.0042 (12)
C1B	0.095 (4)	0.066 (4)	0.073 (4)	−0.004 (3)	0.026 (3)	−0.012 (3)
C2B	0.096 (4)	0.071 (4)	0.076 (4)	0.010 (4)	0.035 (3)	−0.004 (3)
N2B	0.081 (5)	0.065 (5)	0.078 (4)	0.009 (4)	0.032 (4)	0.001 (4)
C3B	0.069 (6)	0.113 (9)	0.131 (8)	0.016 (7)	0.026 (6)	0.003 (8)
C4B	0.099 (7)	0.086 (6)	0.108 (7)	0.012 (6)	0.040 (6)	0.023 (6)
N1B	0.083 (4)	0.064 (4)	0.053 (4)	0.001 (3)	0.014 (3)	0.002 (3)
C5B	0.077 (4)	0.068 (4)	0.054 (3)	−0.006 (4)	0.024 (3)	−0.012 (3)
C6B	0.077 (4)	0.063 (3)	0.065 (3)	−0.001 (3)	0.026 (3)	−0.008 (3)
C7B	0.065 (4)	0.053 (3)	0.050 (3)	0.000 (3)	0.021 (3)	−0.001 (3)
C8B	0.067 (4)	0.054 (3)	0.047 (3)	0.000 (3)	0.019 (3)	0.001 (3)
C9B	0.074 (4)	0.054 (3)	0.052 (4)	0.000 (3)	0.029 (3)	−0.001 (3)
C10B	0.073 (4)	0.053 (4)	0.060 (4)	0.009 (3)	0.034 (4)	−0.007 (3)
C11B	0.064 (4)	0.061 (4)	0.059 (5)	0.011 (3)	0.030 (4)	−0.001 (4)
C12B	0.065 (4)	0.061 (4)	0.051 (4)	−0.006 (3)	0.019 (3)	0.000 (3)
O1B	0.098 (5)	0.066 (4)	0.051 (3)	0.014 (4)	0.005 (3)	−0.002 (3)

### 3-[2-(Dimethylamino)ethyl]-1H-indol-4-ol (Form_II_293K) Geometric parameters (Å, º)

**Table:**

C1—C6	1.509 (6)	C1B—C2B	1.523 (12)
C1—C2	1.530 (6)	C1B—H1C	0.9700
C1—H1A	0.9700	C1B—H1D	0.9700
C1—H1B	0.9700	C2B—N2B	1.463 (13)
C2—N2	1.466 (7)	C2B—H2C	0.9700
C2—H2A	0.9700	C2B—H2D	0.9700
C2—H2B	0.9700	N2B—C4B	1.444 (14)
N2—C4	1.463 (7)	N2B—C3B	1.498 (14)
N2—C3	1.471 (7)	N2B—H1	1.43 (5)
C3—H3A	0.9600	C3B—H3D	0.9600
C3—H3B	0.9600	C3B—H3E	0.9600
C3—H3C	0.9600	C3B—H3F	0.9600
C4—H4A	0.9600	C4B—H4D	0.9600
C4—H4B	0.9600	C4B—H4E	0.9600
C4—H4C	0.9600	C4B—H4F	0.9600
N1—C5	1.350 (7)	N1B—C5B	1.347 (13)
N1—C12	1.370 (6)	N1B—C12B	1.359 (13)
N1—H1N	0.8600	N1B—H1NB	0.8600
C5—C6	1.337 (7)	C5B—C6B	1.368 (13)
C5—H5	0.9300	C5B—H5B	0.9300
C6—C7	1.449 (6)	C6B—C7B	1.472 (12)
C7—C8	1.403 (6)	C7B—C8B	1.407 (12)
C7—C12	1.404 (6)	C7B—C12B	1.416 (13)
C8—O1	1.364 (5)	C8B—O1B	1.333 (11)
C8—C9	1.379 (7)	C8B—C9B	1.374 (12)
C9—C10	1.408 (7)	C9B—C10B	1.369 (13)
C9—H9	0.9300	C9B—H9B	0.9300
C10—C11	1.358 (7)	C10B—C11B	1.340 (13)
C10—H10	0.9300	C10B—H10B	0.9300
C11—C12	1.380 (7)	C11B—C12B	1.397 (13)
C11—H11	0.9300	C11B—H11B	0.9300
O1—H1	1.08 (5)	O1B—H1	1.12 (5)
C1B—C6B	1.521 (12)		
			
C6—C1—C2	114.9 (4)	C2B—C1B—H1C	108.1
C6—C1—H1A	108.5	C6B—C1B—H1D	108.1
C2—C1—H1A	108.5	C2B—C1B—H1D	108.1
C6—C1—H1B	108.5	H1C—C1B—H1D	107.3
C2—C1—H1B	108.5	N2B—C2B—C1B	110.8 (9)
H1A—C1—H1B	107.5	N2B—C2B—H2C	109.5
N2—C2—C1	113.5 (4)	C1B—C2B—H2C	109.5
N2—C2—H2A	108.9	N2B—C2B—H2D	109.5
C1—C2—H2A	108.9	C1B—C2B—H2D	109.5
N2—C2—H2B	108.9	H2C—C2B—H2D	108.1
C1—C2—H2B	108.9	C4B—N2B—C2B	114.7 (13)
H2A—C2—H2B	107.7	C4B—N2B—C3B	109.4 (12)
C4—N2—C2	110.0 (5)	C2B—N2B—C3B	108.7 (12)
C4—N2—C3	109.9 (5)	C4B—N2B—H1	114 (2)
C2—N2—C3	113.9 (6)	C2B—N2B—H1	108.3 (19)
N2—C3—H3A	109.5	C3B—N2B—H1	101 (2)
N2—C3—H3B	109.5	N2B—C3B—H3D	109.5
H3A—C3—H3B	109.5	N2B—C3B—H3E	109.5
N2—C3—H3C	109.5	H3D—C3B—H3E	109.5
H3A—C3—H3C	109.5	N2B—C3B—H3F	109.5
H3B—C3—H3C	109.5	H3D—C3B—H3F	109.5
N2—C4—H4A	109.5	H3E—C3B—H3F	109.5
N2—C4—H4B	109.5	N2B—C4B—H4D	109.5
H4A—C4—H4B	109.5	N2B—C4B—H4E	109.5
N2—C4—H4C	109.5	H4D—C4B—H4E	109.5
H4A—C4—H4C	109.5	N2B—C4B—H4F	109.5
H4B—C4—H4C	109.5	H4D—C4B—H4F	109.5
C5—N1—C12	109.1 (6)	H4E—C4B—H4F	109.5
C5—N1—H1N	125.4	C5B—N1B—C12B	110.4 (12)
C12—N1—H1N	125.4	C5B—N1B—H1NB	124.8
C6—C5—N1	110.7 (5)	C12B—N1B—H1NB	124.8
C6—C5—H5	124.7	N1B—C5B—C6B	112.7 (12)
N1—C5—H5	124.7	N1B—C5B—H5B	123.6
C5—C6—C7	107.0 (5)	C6B—C5B—H5B	123.6
C5—C6—C1	121.4 (5)	C5B—C6B—C7B	102.2 (10)
C7—C6—C1	131.3 (5)	C5B—C6B—C1B	125.6 (11)
C8—C7—C12	119.4 (5)	C7B—C6B—C1B	131.7 (10)
C8—C7—C6	134.9 (5)	C8B—C7B—C12B	113.1 (11)
C12—C7—C6	105.5 (4)	C8B—C7B—C6B	137.3 (12)
O1—C8—C9	118.9 (5)	C12B—C7B—C6B	108.8 (10)
O1—C8—C7	122.9 (5)	O1B—C8B—C9B	115.5 (10)
C9—C8—C7	118.2 (5)	O1B—C8B—C7B	123.5 (11)
C8—C9—C10	120.1 (6)	C9B—C8B—C7B	120.9 (10)
C8—C9—H9	119.9	C10B—C9B—C8B	123.9 (12)
C10—C9—H9	119.9	C10B—C9B—H9B	118.1
C11—C10—C9	122.8 (7)	C8B—C9B—H9B	118.1
C11—C10—H10	118.6	C11B—C10B—C9B	117.7 (14)
C9—C10—H10	118.6	C11B—C10B—H10B	121.2
C10—C11—C12	116.7 (6)	C9B—C10B—H10B	121.2
C10—C11—H11	121.6	C10B—C11B—C12B	119.6 (14)
C12—C11—H11	121.6	C10B—C11B—H11B	120.2
N1—C12—C11	129.5 (6)	C12B—C11B—H11B	120.2
N1—C12—C7	107.7 (5)	N1B—C12B—C11B	129.8 (13)
C11—C12—C7	122.6 (6)	N1B—C12B—C7B	105.7 (11)
C8—O1—H1	109 (2)	C11B—C12B—C7B	124.4 (13)
C6B—C1B—C2B	116.7 (8)	C8B—O1B—H1	111 (2)
C6B—C1B—H1C	108.1		
			
C6—C1—C2—N2	−84.6 (6)	C6B—C1B—C2B—N2B	83.2 (13)
C1—C2—N2—C4	160.7 (5)	C1B—C2B—N2B—C4B	76.8 (14)
C1—C2—N2—C3	−75.4 (6)	C1B—C2B—N2B—C3B	−160.4 (12)
C12—N1—C5—C6	−1.3 (8)	C12B—N1B—C5B—C6B	1.6 (15)
N1—C5—C6—C7	1.9 (7)	N1B—C5B—C6B—C7B	−3.9 (10)
N1—C5—C6—C1	−172.9 (5)	N1B—C5B—C6B—C1B	168.7 (12)
C2—C1—C6—C5	−117.6 (6)	C2B—C1B—C6B—C5B	130.1 (10)
C2—C1—C6—C7	69.1 (8)	C2B—C1B—C6B—C7B	−59.5 (17)
C5—C6—C7—C8	−177.2 (8)	C5B—C6B—C7B—C8B	173 (2)
C1—C6—C7—C8	−3.3 (13)	C1B—C6B—C7B—C8B	1 (3)
C5—C6—C7—C12	−1.6 (9)	C5B—C6B—C7B—C12B	4.8 (19)
C1—C6—C7—C12	172.3 (7)	C1B—C6B—C7B—C12B	−167.2 (18)
C12—C7—C8—O1	−177.9 (8)	C12B—C7B—C8B—O1B	173.0 (19)
C6—C7—C8—O1	−2.8 (12)	C6B—C7B—C8B—O1B	5 (3)
C12—C7—C8—C9	−0.4 (11)	C12B—C7B—C8B—C9B	−3 (3)
C6—C7—C8—C9	174.7 (8)	C6B—C7B—C8B—C9B	−171.8 (19)
O1—C8—C9—C10	178.6 (7)	O1B—C8B—C9B—C10B	−175.0 (17)
C7—C8—C9—C10	1.1 (10)	C7B—C8B—C9B—C10B	2 (2)
C8—C9—C10—C11	−3.6 (14)	C8B—C9B—C10B—C11B	4 (3)
C9—C10—C11—C12	5.1 (16)	C9B—C10B—C11B—C12B	−8 (3)
C5—N1—C12—C11	174.9 (11)	C5B—N1B—C12B—C11B	−173 (3)
C5—N1—C12—C7	0.2 (10)	C5B—N1B—C12B—C7B	2 (2)
C10—C11—C12—N1	−178.3 (12)	C10B—C11B—C12B—N1B	−180 (3)
C10—C11—C12—C7	−4.4 (16)	C10B—C11B—C12B—C7B	6 (4)
C8—C7—C12—N1	177.3 (8)	C8B—C7B—C12B—N1B	−175.7 (18)
C6—C7—C12—N1	0.8 (10)	C6B—C7B—C12B—N1B	−4 (3)
C8—C7—C12—C11	2.2 (15)	C8B—C7B—C12B—C11B	0 (4)
C6—C7—C12—C11	−174.3 (10)	C6B—C7B—C12B—C11B	171 (2)

### 3-[2-(Dimethylamino)ethyl]-1H-indol-4-ol (Form_II_293K) Hydrogen-bond geometry (Å, º)

**Table:**

*D*—H···*A*	*D*—H	H···*A*	*D*···*A*	*D*—H···*A*
N1—H1*N*···O1^i^	0.86	1.99	2.823 (8)	164
N1*B*—H1*NB*···O1*B*^i^	0.86	2.14	2.984 (18)	169
O1—H1···N2	1.08 (5)	1.54 (5)	2.584 (8)	161 (4)
O1*B*—H1···N2*B*	1.12 (5)	1.43 (5)	2.52 (2)	162 (4)

Symmetry code: (i) *x*−1/2, −*y*+3/2, *z*−1/2.

### (Form_I_150K) Crystal data

**Table:**

C_12_H_16_N_2_O	*F*(000) = 440
*M**_r_* = 204.27	*D*_x_ = 1.233 Mg m^−^^3^
Monoclinic, *P*2_1_/*c*	Mo *K*α radiation, λ = 0.71073 Å
*a* = 10.4041 (4) Å	Cell parameters from 9931 reflections
*b* = 8.5286 (3) Å	θ = 3.1–33.1°
*c* = 12.4087 (4) Å	µ = 0.08 mm^−^^1^
β = 92.5663 (19)°	*T* = 150 K
*V* = 1099.95 (7) Å^3^	Plate, colourless
*Z* = 4	0.43 × 0.29 × 0.06 mm

### (Form_I_150K) Data collection

**Table:**

Bruker AXS D8 Quest diffractometer	4199 independent reflections
Radiation source: fine focus sealed tube X-ray source	3116 reflections with *I* > 2σ(*I*)
Triumph curved graphite crystal monochromator	*R*_int_ = 0.051
Detector resolution: 7.4074 pixels mm^-1^	θ_max_ = 33.2°, θ_min_ = 2.9°
ω and phi scans	*h* = −16→16
Absorption correction: multi-scan (SADABS 2016/2; Krause *et al.*, 2015)	*k* = −13→13
*T*_min_ = 0.663, *T*_max_ = 0.747	*l* = −19→18
33575 measured reflections	

### (Form_I_150K) Refinement

**Table:**

Refinement on *F*^2^	Primary atom site location: dual
Least-squares matrix: full	Secondary atom site location: difference Fourier map
*R*[*F*^2^ > 2σ(*F*^2^)] = 0.046	Hydrogen site location: mixed
*wR*(*F*^2^) = 0.138	H atoms treated by a mixture of independent and constrained refinement
*S* = 1.05	*w* = 1/[σ^2^(*F*_o_^2^) + (0.0713*P*)^2^ + 0.1621*P*] where *P* = (*F*_o_^2^ + 2*F*_c_^2^)/3
4199 reflections	(Δ/σ)_max_ < 0.001
146 parameters	Δρ_max_ = 0.35 e Å^−^^3^
0 restraints	Δρ_min_ = −0.24 e Å^−^^3^

### (Form_I_150K) Special details

**Table:**

Geometry. All esds (except the esd in the dihedral angle between two l.s. planes) are estimated using the full covariance matrix. The cell esds are taken into account individually in the estimation of esds in distances, angles and torsion angles; correlations between esds in cell parameters are only used when they are defined by crystal symmetry. An approximate (isotropic) treatment of cell esds is used for estimating esds involving l.s. planes.
Refinement. The atom naming scheme was adopted from an earlier determination of this structure (T. J. Petcher, H. P. Weber, J. C. S. Perkin Trans. 2, 1974, 946-948, https://doi.org/10.1039/P29740000946). Positions and isotropic displacement parameters of acidic H atoms (O-H and N-H) were freely refined.

### (Form_I_150K) Fractional atomic coordinates and isotropic or equivalent isotropic displacement parameters (Å^2^)

**Table:**

	*x*	*y*	*z*	*U*_iso_*/*U*_eq_	
O1	0.70597 (7)	0.47588 (8)	0.47509 (5)	0.03151 (16)	
H1O	0.7335 (17)	0.592 (2)	0.4841 (14)	0.071 (5)*	
N1	0.64472 (10)	0.13425 (11)	0.18670 (7)	0.0385 (2)	
H1N	0.6505 (15)	0.0898 (19)	0.1202 (13)	0.056 (4)*	
N2	0.24988 (8)	0.22499 (9)	0.49394 (6)	0.02849 (16)	
C1	0.47558 (9)	0.23991 (11)	0.43345 (7)	0.02952 (18)	
H1A	0.472877	0.351903	0.454214	0.035*	
H1B	0.513277	0.180568	0.495733	0.035*	
C5	0.54133 (11)	0.12554 (12)	0.25174 (8)	0.0350 (2)	
H5	0.467292	0.062248	0.237856	0.042*	
C6	0.56084 (9)	0.22194 (10)	0.33987 (7)	0.02783 (18)	
C7	0.68352 (9)	0.29695 (10)	0.32686 (7)	0.02674 (18)	
C8	0.75460 (9)	0.41272 (10)	0.38446 (7)	0.02762 (18)	
C9	0.87124 (10)	0.46122 (13)	0.34568 (9)	0.0357 (2)	
H9	0.920818	0.537842	0.384468	0.043*	
C10	0.91725 (11)	0.39842 (14)	0.24952 (9)	0.0415 (3)	
H10	0.997389	0.433933	0.224968	0.050*	
C11	0.84970 (11)	0.28788 (14)	0.19068 (9)	0.0403 (3)	
H11	0.881122	0.246287	0.125900	0.048*	
C12	0.73231 (10)	0.23859 (11)	0.22993 (7)	0.0324 (2)	
C2	0.33887 (9)	0.18255 (12)	0.40912 (8)	0.0326 (2)	
H2A	0.306539	0.227858	0.339629	0.039*	
H2B	0.339916	0.067070	0.401086	0.039*	
C3	0.11742 (11)	0.18671 (17)	0.45855 (11)	0.0477 (3)	
H3A	0.095506	0.240663	0.390463	0.072*	
H3B	0.058665	0.220643	0.513544	0.072*	
H3C	0.109444	0.073191	0.447973	0.072*	
C4	0.28086 (12)	0.14338 (14)	0.59591 (9)	0.0417 (2)	
H4A	0.274408	0.029888	0.584536	0.063*	
H4B	0.220221	0.175593	0.649912	0.063*	
H4C	0.368648	0.170138	0.621391	0.063*	

### (Form_I_150K) Atomic displacement parameters (Å^2^)

**Table:**

	*U*^11^	*U*^22^	*U*^33^	*U*^12^	*U*^13^	*U*^23^
O1	0.0440 (4)	0.0254 (3)	0.0263 (3)	−0.0044 (3)	0.0143 (3)	−0.0015 (2)
N1	0.0508 (5)	0.0386 (5)	0.0265 (4)	0.0103 (4)	0.0070 (3)	−0.0076 (3)
N2	0.0294 (4)	0.0283 (4)	0.0280 (3)	−0.0019 (3)	0.0039 (3)	−0.0006 (3)
C1	0.0325 (4)	0.0296 (4)	0.0269 (4)	−0.0009 (3)	0.0058 (3)	−0.0038 (3)
C5	0.0437 (5)	0.0319 (5)	0.0295 (4)	0.0051 (4)	0.0025 (4)	−0.0067 (4)
C6	0.0345 (4)	0.0245 (4)	0.0249 (4)	0.0047 (3)	0.0052 (3)	−0.0014 (3)
C7	0.0340 (4)	0.0244 (4)	0.0224 (3)	0.0081 (3)	0.0079 (3)	0.0028 (3)
C8	0.0343 (4)	0.0249 (4)	0.0244 (4)	0.0049 (3)	0.0093 (3)	0.0043 (3)
C9	0.0355 (5)	0.0361 (5)	0.0364 (5)	0.0024 (4)	0.0119 (4)	0.0043 (4)
C10	0.0373 (5)	0.0480 (6)	0.0408 (5)	0.0100 (4)	0.0186 (4)	0.0091 (4)
C11	0.0448 (6)	0.0457 (6)	0.0318 (5)	0.0170 (5)	0.0174 (4)	0.0041 (4)
C12	0.0422 (5)	0.0304 (4)	0.0251 (4)	0.0125 (4)	0.0094 (3)	0.0015 (3)
C2	0.0334 (4)	0.0346 (5)	0.0301 (4)	−0.0006 (4)	0.0033 (3)	−0.0078 (4)
C3	0.0306 (5)	0.0634 (8)	0.0492 (6)	−0.0026 (5)	0.0012 (4)	−0.0151 (6)
C4	0.0452 (6)	0.0420 (6)	0.0383 (5)	−0.0024 (5)	0.0067 (4)	0.0142 (4)

### (Form_I_150K) Geometric parameters (Å, º)

**Table:**

O1—C8	1.3645 (10)	C7—C12	1.4165 (12)
O1—H1O	1.038 (19)	C8—C9	1.3884 (13)
N1—C12	1.3662 (15)	C9—C10	1.4110 (15)
N1—C5	1.3755 (14)	C9—H9	0.9500
N1—H1N	0.913 (16)	C10—C11	1.3674 (18)
N2—C3	1.4644 (14)	C10—H10	0.9500
N2—C4	1.4672 (13)	C11—C12	1.3995 (15)
N2—C2	1.4776 (12)	C11—H11	0.9500
C1—C6	1.5004 (12)	C2—H2A	0.9900
C1—C2	1.5215 (14)	C2—H2B	0.9900
C1—H1A	0.9900	C3—H3A	0.9800
C1—H1B	0.9900	C3—H3B	0.9800
C5—C6	1.3759 (13)	C3—H3C	0.9800
C5—H5	0.9500	C4—H4A	0.9800
C6—C7	1.4432 (13)	C4—H4B	0.9800
C7—C8	1.4090 (13)	C4—H4C	0.9800
			
C8—O1—H1O	110.8 (10)	C11—C10—C9	121.84 (10)
C12—N1—C5	109.28 (8)	C11—C10—H10	119.1
C12—N1—H1N	123.8 (10)	C9—C10—H10	119.1
C5—N1—H1N	126.4 (10)	C10—C11—C12	117.19 (9)
C3—N2—C4	108.68 (9)	C10—C11—H11	121.4
C3—N2—C2	110.07 (8)	C12—C11—H11	121.4
C4—N2—C2	112.18 (8)	N1—C12—C11	129.50 (9)
C6—C1—C2	113.17 (8)	N1—C12—C7	107.71 (9)
C6—C1—H1A	108.9	C11—C12—C7	122.78 (10)
C2—C1—H1A	108.9	N2—C2—C1	112.95 (8)
C6—C1—H1B	108.9	N2—C2—H2A	109.0
C2—C1—H1B	108.9	C1—C2—H2A	109.0
H1A—C1—H1B	107.8	N2—C2—H2B	109.0
N1—C5—C6	110.04 (10)	C1—C2—H2B	109.0
N1—C5—H5	125.0	H2A—C2—H2B	107.8
C6—C5—H5	125.0	N2—C3—H3A	109.5
C5—C6—C7	106.01 (8)	N2—C3—H3B	109.5
C5—C6—C1	127.22 (9)	H3A—C3—H3B	109.5
C7—C6—C1	126.71 (8)	N2—C3—H3C	109.5
C8—C7—C12	118.49 (8)	H3A—C3—H3C	109.5
C8—C7—C6	134.47 (8)	H3B—C3—H3C	109.5
C12—C7—C6	106.95 (8)	N2—C4—H4A	109.5
O1—C8—C9	122.01 (9)	N2—C4—H4B	109.5
O1—C8—C7	119.29 (8)	H4A—C4—H4B	109.5
C9—C8—C7	118.69 (8)	N2—C4—H4C	109.5
C8—C9—C10	120.99 (11)	H4A—C4—H4C	109.5
C8—C9—H9	119.5	H4B—C4—H4C	109.5
C10—C9—H9	119.5		
			
C12—N1—C5—C6	−1.19 (12)	C7—C8—C9—C10	1.12 (15)
N1—C5—C6—C7	1.10 (11)	C8—C9—C10—C11	−0.03 (17)
N1—C5—C6—C1	−176.13 (9)	C9—C10—C11—C12	−0.17 (17)
C2—C1—C6—C5	−19.25 (14)	C5—N1—C12—C11	−178.23 (10)
C2—C1—C6—C7	164.07 (9)	C5—N1—C12—C7	0.76 (11)
C5—C6—C7—C8	175.89 (10)	C10—C11—C12—N1	178.11 (10)
C1—C6—C7—C8	−6.86 (16)	C10—C11—C12—C7	−0.75 (16)
C5—C6—C7—C12	−0.61 (10)	C8—C7—C12—N1	−177.24 (8)
C1—C6—C7—C12	176.64 (8)	C6—C7—C12—N1	−0.08 (10)
C12—C7—C8—O1	176.77 (8)	C8—C7—C12—C11	1.83 (14)
C6—C7—C8—O1	0.57 (15)	C6—C7—C12—C11	178.99 (9)
C12—C7—C8—C9	−1.96 (13)	C3—N2—C2—C1	171.85 (9)
C6—C7—C8—C9	−178.15 (10)	C4—N2—C2—C1	−67.00 (11)
O1—C8—C9—C10	−177.57 (9)	C6—C1—C2—N2	−170.10 (8)

### (Form_I_150K) Hydrogen-bond geometry (Å, º)

**Table:**

*D*—H···*A*	*D*—H	H···*A*	*D*···*A*	*D*—H···*A*
O1—H1*O*···N2^i^	1.038 (19)	1.590 (19)	2.6175 (10)	169.5 (16)
N1—H1*N*···O1^ii^	0.913 (16)	1.995 (17)	2.8867 (11)	165.0 (15)

Symmetry codes: (i) −*x*+1, −*y*+1, −*z*+1; (ii) *x*, −*y*+1/2, *z*−1/2.

### (Form_I_300K) Crystal data

**Table:**

C_12_H_16_N_2_O	*F*(000) = 440
*M**_r_* = 204.27	*D*_x_ = 1.202 Mg m^−^^3^
Monoclinic, *P*2_1_/*c*	Mo *K*α radiation, λ = 0.71073 Å
*a* = 10.6228 (5) Å	Cell parameters from 7001 reflections
*b* = 8.4984 (4) Å	θ = 3.1–30.2°
*c* = 12.5073 (5) Å	µ = 0.08 mm^−^^1^
β = 90.807 (2)°	*T* = 300 K
*V* = 1129.01 (9) Å^3^	Plate, colourless
*Z* = 4	0.43 × 0.29 × 0.06 mm

### (Form_I_300K) Data collection

**Table:**

Bruker AXS D8 Quest diffractometer	4323 independent reflections
Radiation source: fine focus sealed tube X-ray source	2421 reflections with *I* > 2σ(*I*)
Triumph curved graphite crystal monochromator	*R*_int_ = 0.066
Detector resolution: 7.4074 pixels mm^-1^	θ_max_ = 33.2°, θ_min_ = 2.9°
ω and phi scans	*h* = −16→16
Absorption correction: multi-scan (SADABS 2016/2; Krause *et al.*, 2015)	*k* = −13→13
*T*_min_ = 0.663, *T*_max_ = 0.747	*l* = −19→18
34680 measured reflections	

### (Form_I_300K) Refinement

**Table:**

Refinement on *F*^2^	Primary atom site location: isomorphous structure methods
Least-squares matrix: full	Secondary atom site location: difference Fourier map
*R*[*F*^2^ > 2σ(*F*^2^)] = 0.052	Hydrogen site location: mixed
*wR*(*F*^2^) = 0.172	H atoms treated by a mixture of independent and constrained refinement
*S* = 1.03	*w* = 1/[σ^2^(*F*_o_^2^) + (0.0782*P*)^2^ + 0.1036*P*] where *P* = (*F*_o_^2^ + 2*F*_c_^2^)/3
4323 reflections	(Δ/σ)_max_ < 0.001
146 parameters	Δρ_max_ = 0.21 e Å^−^^3^
0 restraints	Δρ_min_ = −0.19 e Å^−^^3^

### (Form_I_300K) Special details

**Table:**

Geometry. All esds (except the esd in the dihedral angle between two l.s. planes) are estimated using the full covariance matrix. The cell esds are taken into account individually in the estimation of esds in distances, angles and torsion angles; correlations between esds in cell parameters are only used when they are defined by crystal symmetry. An approximate (isotropic) treatment of cell esds is used for estimating esds involving l.s. planes.
Refinement. The structure was solved from its 150 K analogue by isomorphous replacement. The atom naming scheme was adopted from an earlier determination of this structure (T. J. Petcher, H. P. Weber, J. C. S. Perkin Trans. 2, 1974, 946-948, https://doi.org/10.1039/P29740000946). Positions and isotropic displacement parameters of acidic H atoms (O-H and N-H) were freely refined.

### (Form_I_300K) Fractional atomic coordinates and isotropic or equivalent isotropic displacement parameters (Å^2^)

**Table:**

	*x*	*y*	*z*	*U*_iso_*/*U*_eq_	
O1	0.69993 (9)	0.47438 (10)	0.46870 (6)	0.0560 (2)	
H1O	0.7313 (19)	0.588 (3)	0.4810 (15)	0.103 (6)*	
N1	0.64455 (13)	0.13744 (16)	0.18151 (10)	0.0705 (4)	
H1N	0.6508 (18)	0.095 (2)	0.1144 (16)	0.093 (6)*	
N2	0.25313 (10)	0.22804 (13)	0.49141 (9)	0.0552 (3)	
C1	0.47404 (11)	0.24181 (16)	0.42811 (10)	0.0541 (3)	
H1A	0.473533	0.351254	0.450111	0.065*	
H1B	0.507116	0.180151	0.487413	0.065*	
C5	0.54246 (14)	0.12965 (17)	0.24671 (11)	0.0639 (4)	
H5	0.471156	0.068880	0.233330	0.077*	
C6	0.55999 (12)	0.22397 (14)	0.33453 (9)	0.0502 (3)	
C7	0.68108 (11)	0.29721 (13)	0.32125 (8)	0.0464 (3)	
C8	0.75013 (11)	0.41104 (14)	0.37825 (9)	0.0483 (3)	
C9	0.86570 (13)	0.45722 (18)	0.33999 (12)	0.0636 (3)	
H9	0.913117	0.531082	0.377831	0.076*	
C10	0.91262 (14)	0.3942 (2)	0.24471 (13)	0.0739 (4)	
H10	0.990900	0.427232	0.221063	0.089*	
C11	0.84729 (15)	0.2863 (2)	0.18597 (12)	0.0709 (4)	
H11	0.879046	0.245610	0.122822	0.085*	
C12	0.73034 (13)	0.23927 (16)	0.22452 (10)	0.0567 (3)	
C2	0.34100 (13)	0.19072 (18)	0.40446 (11)	0.0620 (3)	
H2A	0.311821	0.241790	0.339330	0.074*	
H2B	0.340035	0.078053	0.392046	0.074*	
C3	0.12307 (15)	0.2040 (3)	0.45468 (18)	0.1042 (7)	
H3A	0.105329	0.272151	0.395138	0.156*	
H3B	0.066678	0.227621	0.511797	0.156*	
H3C	0.111940	0.096468	0.433020	0.156*	
C4	0.27790 (19)	0.1351 (2)	0.58787 (15)	0.0928 (6)	
H4A	0.263611	0.025763	0.572681	0.139*	
H4B	0.222611	0.168533	0.643515	0.139*	
H4C	0.363726	0.149990	0.610712	0.139*	

### (Form_I_300K) Atomic displacement parameters (Å^2^)

**Table:**

	*U*^11^	*U*^22^	*U*^33^	*U*^12^	*U*^13^	*U*^23^
O1	0.0711 (6)	0.0524 (5)	0.0450 (4)	−0.0088 (4)	0.0212 (4)	−0.0048 (3)
N1	0.0868 (9)	0.0741 (8)	0.0509 (6)	0.0118 (6)	0.0123 (6)	−0.0191 (6)
N2	0.0498 (5)	0.0578 (6)	0.0582 (6)	−0.0052 (4)	0.0087 (4)	−0.0046 (5)
C1	0.0550 (7)	0.0573 (7)	0.0502 (6)	−0.0007 (5)	0.0100 (5)	−0.0069 (5)
C5	0.0720 (8)	0.0631 (8)	0.0568 (7)	0.0026 (6)	0.0053 (6)	−0.0161 (6)
C6	0.0567 (6)	0.0473 (6)	0.0467 (6)	0.0075 (5)	0.0080 (5)	−0.0035 (5)
C7	0.0546 (6)	0.0456 (5)	0.0393 (5)	0.0132 (5)	0.0115 (4)	0.0027 (4)
C8	0.0538 (6)	0.0504 (6)	0.0412 (5)	0.0077 (5)	0.0128 (4)	0.0061 (4)
C9	0.0558 (7)	0.0719 (8)	0.0635 (8)	0.0007 (6)	0.0169 (6)	0.0038 (6)
C10	0.0583 (8)	0.0934 (11)	0.0709 (9)	0.0135 (7)	0.0281 (7)	0.0103 (8)
C11	0.0722 (9)	0.0856 (10)	0.0557 (7)	0.0240 (8)	0.0278 (6)	0.0008 (7)
C12	0.0669 (7)	0.0587 (7)	0.0449 (6)	0.0190 (6)	0.0142 (5)	−0.0003 (5)
C2	0.0589 (7)	0.0664 (8)	0.0609 (7)	−0.0042 (6)	0.0082 (6)	−0.0170 (6)
C3	0.0523 (8)	0.1488 (19)	0.1117 (15)	−0.0096 (10)	0.0041 (9)	−0.0549 (14)
C4	0.0979 (13)	0.0873 (12)	0.0940 (12)	0.0032 (10)	0.0288 (10)	0.0360 (10)

### (Form_I_300K) Geometric parameters (Å, º)

**Table:**

O1—C8	1.3678 (13)	C7—C12	1.4135 (15)
O1—H1O	1.03 (2)	C8—C9	1.3809 (17)
N1—C12	1.362 (2)	C9—C10	1.405 (2)
N1—C5	1.3676 (19)	C9—H9	0.9300
N1—H1N	0.92 (2)	C10—C11	1.359 (2)
N2—C4	1.463 (2)	C10—H10	0.9300
N2—C3	1.4640 (19)	C11—C12	1.397 (2)
N2—C2	1.4776 (16)	C11—H11	0.9300
C1—C6	1.5022 (15)	C2—H2A	0.9700
C1—C2	1.5038 (18)	C2—H2B	0.9700
C1—H1A	0.9700	C3—H3A	0.9600
C1—H1B	0.9700	C3—H3B	0.9600
C5—C6	1.3703 (17)	C3—H3C	0.9600
C5—H5	0.9300	C4—H4A	0.9600
C6—C7	1.4407 (17)	C4—H4B	0.9600
C7—C8	1.4026 (18)	C4—H4C	0.9600
			
C8—O1—H1O	111.3 (11)	C11—C10—C9	122.10 (13)
C12—N1—C5	109.09 (11)	C11—C10—H10	118.9
C12—N1—H1N	123.6 (12)	C9—C10—H10	118.9
C5—N1—H1N	126.6 (12)	C10—C11—C12	117.14 (12)
C4—N2—C3	109.96 (15)	C10—C11—H11	121.4
C4—N2—C2	112.52 (12)	C12—C11—H11	121.4
C3—N2—C2	110.01 (12)	N1—C12—C11	129.70 (13)
C6—C1—C2	113.42 (11)	N1—C12—C7	107.80 (12)
C6—C1—H1A	108.9	C11—C12—C7	122.50 (14)
C2—C1—H1A	108.9	N2—C2—C1	113.26 (11)
C6—C1—H1B	108.9	N2—C2—H2A	108.9
C2—C1—H1B	108.9	C1—C2—H2A	108.9
H1A—C1—H1B	107.7	N2—C2—H2B	108.9
N1—C5—C6	110.47 (13)	C1—C2—H2B	108.9
N1—C5—H5	124.8	H2A—C2—H2B	107.7
C6—C5—H5	124.8	N2—C3—H3A	109.5
C5—C6—C7	105.78 (11)	N2—C3—H3B	109.5
C5—C6—C1	127.32 (12)	H3A—C3—H3B	109.5
C7—C6—C1	126.84 (11)	N2—C3—H3C	109.5
C8—C7—C12	118.56 (11)	H3A—C3—H3C	109.5
C8—C7—C6	134.49 (10)	H3B—C3—H3C	109.5
C12—C7—C6	106.86 (11)	N2—C4—H4A	109.5
O1—C8—C9	122.25 (12)	N2—C4—H4B	109.5
O1—C8—C7	118.99 (10)	H4A—C4—H4B	109.5
C9—C8—C7	118.75 (11)	N2—C4—H4C	109.5
C8—C9—C10	120.89 (15)	H4A—C4—H4C	109.5
C8—C9—H9	119.6	H4B—C4—H4C	109.5
C10—C9—H9	119.6		
			
C12—N1—C5—C6	−0.99 (17)	C7—C8—C9—C10	1.3 (2)
N1—C5—C6—C7	1.03 (15)	C8—C9—C10—C11	0.2 (2)
N1—C5—C6—C1	−176.28 (12)	C9—C10—C11—C12	−0.3 (2)
C2—C1—C6—C5	−20.34 (19)	C5—N1—C12—C11	−179.00 (15)
C2—C1—C6—C7	162.90 (12)	C5—N1—C12—C7	0.52 (15)
C5—C6—C7—C8	175.62 (13)	C10—C11—C12—N1	178.25 (14)
C1—C6—C7—C8	−7.1 (2)	C10—C11—C12—C7	−1.2 (2)
C5—C6—C7—C12	−0.69 (13)	C8—C7—C12—N1	−176.89 (11)
C1—C6—C7—C12	176.64 (11)	C6—C7—C12—N1	0.12 (14)
C12—C7—C8—O1	176.31 (10)	C8—C7—C12—C11	2.67 (19)
C6—C7—C8—O1	0.3 (2)	C6—C7—C12—C11	179.68 (12)
C12—C7—C8—C9	−2.62 (17)	C4—N2—C2—C1	−68.20 (16)
C6—C7—C8—C9	−178.60 (13)	C3—N2—C2—C1	168.82 (15)
O1—C8—C9—C10	−177.63 (12)	C6—C1—C2—N2	−172.27 (11)

### (Form_I_300K) Hydrogen-bond geometry (Å, º)

**Table:**

*D*—H···*A*	*D*—H	H···*A*	*D*···*A*	*D*—H···*A*
O1—H1*O*···N2^i^	1.03 (2)	1.61 (2)	2.6242 (14)	166.6 (18)
N1—H1*N*···O1^ii^	0.92 (2)	1.99 (2)	2.8944 (14)	167.9 (18)

Symmetry codes: (i) −*x*+1, −*y*+1, −*z*+1; (ii) *x*, −*y*+1/2, *z*−1/2.

### The unit-cell parameters of Form I as a function of temperature. Axes in [Å], angle in [°], volume in [Å^3^]. Alpha and gamma angles are 90° (monoclinic crystal system). Estimated standard deviations are given below each value.

**Table:**

	a-axis	b-axis	c-axis	beta	Volume
150	10.4055	8.5247	12.4170	92.6122	1100.296
	0.0004	0.0003	0.0004	0.0019	0.093
170	10.4506	8.5219	12.4289	92.3237	1106.001
	0.0010	0.0007	0.0011	0.0029	0.278
190	10.4863	8.5145	12.4417	92.0707	1110.171
	0.0011	0.0007	0.0012	0.0031	0.293
210	10.5185	8.5072	12.4516	91.7817	1113.659
	0.0010	0.0006	0.0011	0.0028	0.262
230	10.5462	8.5009	12.4634	91.5068	1116.985
	0.0011	0.0008	0.0013	0.0032	0.318
250	10.5728	8.4975	12.4790	91.3072	1120.845
	0.0011	0.0008	0.0013	0.0031	0.315
270	10.5897	8.4935	12.4886	91.0658	1123.084
	0.0009	0.0006	0.0010	0.0026	0.243
290	10.6137	8.4937	12.4994	90.9311	1126.671
	0.0012	0.0008	0.0014	0.0033	0.337
300	10.6208	8.4943	12.5066	90.8366	1128.181
	0.0005	0.0004	0.0005	0.0024	0.104

### Relative changes of unit cell parameter as a function of temperature. Values relative to the 150 K number.

**Table:**

	a-axis	b-axis	c-axis	Volume
150	1.00	1.00	1.00	1.00
170	1.0043	0.9997	1.0010	1.0052
190	1.0078	0.9988	1.0020	1.0090
210	1.0109	0.9979	1.0028	1.0121
230	1.0135	0.9972	1.0037	1.0152
250	1.0161	0.9968	1.0050	1.0187
270	1.0177	0.9963	1.0058	1.0207
290	1.0200	0.9964	1.0066	1.0240
300	1.0207	0.9964	1.0072	1.0253
